# The impact of COVID-19 pandemic on the cardiology services in Northern Jordan

**DOI:** 10.1016/j.amsu.2020.11.046

**Published:** 2020-11-19

**Authors:** Rasheed Ibdah, Sukaina Rawashdeh, Abdullah Al-Kasasbeh, Mahmoud Albalas, Nail A. Obeidat, Nasr Alrabadi

**Affiliations:** a Division of Cardiology, Department of Internal Medicine, Faculty of Medicine, Jordan University of Science and Technology, 22110, Jordan; b Department of General and Special Surgery, Faculty of Medicine, Hashemite University, Jordan; c Department of Obstetrics and Gynecology, Faculty of Medicine, Jordan University of Science and Technology, 22110, Jordan; d Department of Pharmacology, Faculty of Medicine, Jordan University of Science and Technology, 22110, Jordan

## Abstract

The COVID-19 pandemic, caused by the coronavirus SARS-CoV-2, has rapidly spread worldwide starting from China in late 2019. The first case in Jordan was reported on March 2, 2020. The Jordanian government made many transformations to address this crisis. As we are the only referral cardiology center for percutaneous coronary intervention (PCI) in the north of Jordan, we made multiple adjustments to confront COVID-19 challenges. We emphasize that there is an urgent need to update all procedures and therapeutic activities that are performed in the Cath-Lab to minimize the risks for both the patients and the health care providers during the pandemic of COVID-19.

## 1 Introduction

The pneumonia of unknown cause that was detected in Wuhan; China first was reported to the WHO Country Office on December 31, 2019 [1]. Shortly later, this was recognized as the first case of infection with the coronavirus SARS-CoV-2 and the first case of COVID-19. In Jordan, the first case of COVID-19 was reported on March 2, 2020. The total number of COVID-19 confirmed cases in Jordan as of the November 3, 2020, was 81,743, with 913 deaths [2]. In response to that, the Jordanian government made many critical changes to address this crisis. Starting from March 14, 2020, Jordan declared a state of emergency, and a lockdown law was activated where even the schools and the universities were closed, as well as, the mosques, the churches, and the borders.

Immediately after that, the Ministry of Health assigned two major hospitals in Jordan as referral centers for patients with COVID-19. One of which is King Abdullah University Hospital (KAUH) in the north of Jordan. KAUH is a large tertiary center with 683 beds, expandable to 800 beds during emergencies [3]. It is a teaching hospital affiliated with Jordan University of Science and Technology (JUST)/School of Medicine, and it is one of the leading medical schools in Jordan and the Middle East [4]. KAUH is now the only Catheterization referral center for COVID-19 cases in the north of Jordan, serving around 3 million people.

As the government announced the emergency state and the lockdown, the KAUH outpatient clinics were closed, and all elective procedures and surgeries were rescheduled. Considered as the only referral cardiology center for Percutaneous Coronary Intervention (PCI) in the north of Jordan, the Department of internal medicine/Cardiology Division at JUST/KAUH has made multiple adjustments to confront the COVID-19 challenges. Some of those adjustments were related to the hospital general recommendations and others were specifically related to Cath-Lab.

## 2 Adjustments related to the hospital general recommendations

After closing the regular services of the hospital clinics, the hospital system kept some clinic activities open to refill regular medical prescriptions for chronic diseases. A group of volunteers from our medical school contributed with enthusiasm to this process by delivering drugs to the patients unable to reach the hospital due to the lockdown. Patients with acute cardiac problems were directed by official authorities and the local media, to visit the emergency department (ED), where an in-house cardiology team is available 24/7 to evaluate these patients. In general, in-patients were kept in the hospital for the possible shortest stay without affecting their medical care to decrease their chance of getting hospital-acquired COVID-19 and to protect as possible their health care providers.

On the other hand, those patients with non-urgent chronic cardiovascular diseases who have questions about their medical conditions could reach their assigned physicians by telephone or WhatsApp. Those whose problems were not solved over the phone were advised to visit the ED for further evaluation. In this regard, it is important to highlight that the most frequently discussed issues with patients were the need to adjust their anti-hypertensive medications, the heart failure medications, and the anticoagulants. As well, patients with symptoms suspicious of the acute coronary syndrome (ACS) were advised to visit the emergency department. Moreover, the number of cardiology patients who visited the Cath-Lab during the lockdown period was approximately 1/3 the regular number of patients who used to visit before and after the lockdown period.

## 3 Adjustments specifically related to the Cath-Lab

Special considerations were applied to patients with cardiovascular diseases who develop COVID‐19 as they have a higher risk of mortality [5]. However, it is essential to emphasize that most patients who need cardiovascular care were not infected with this novel coronavirus (Currently, less than 10% (2.5–7%) of our patients are tested positive for COVID-19) As we prepare for the care of patients with COVID‐19‐related illnesses, we also need to ensure that the overall patients population continues to benefit from cardiovascular medical advancements.

COVID-19 is a highly contagious disease, therefore, invasive treatments for coronary artery disease became riskier. Therefore, different cardiac societies in the world have published their recommendations for the management of patients with coronary artery diseases to balance between the benefits of interventions and the risk of COVID-19 infection. In our hospital, these instructions mainly pointed toward the following: to avoid elective cases, to prevent entering suspected patients with COVID-19 to the catheterization laboratory as much as possible (while finding them an alternative isolated facility), to prepare the team with leading personal protective equipment (PPE) when a percutaneous coronary intervention (PCI) is necessary, and to perform the procedure in an isolated room for each patient. It was also emphasized that primary PCI was considered immediately, without waiting for the COVID-19-PCR test results and after considering all possible protective measures, in all ST-elevation myocardial infarction (STEMI) cases presenting to our PCI center or STEMI patients referred from other primary hospitals with either contraindication thrombolysis or failed thrombolytic therapy for rescue PCI.

Finally, a list of recommendations was set by our medical teams based on the international guidelines to provide the ACS patients with the maximum benefits and the best possible practice without compromising their safety and the safety of the hospital staff during the use of the catheterization laboratory.

## 4 List of practical recommendations

1. All elective procedures should be postponed. This step aims to save resources and decrease patients' exposure to the hospital environment where the chance to get infected with COVID-19 might be higher than anywhere else besides reducing the risk of staff to acquire this potentially severe infection.
2. All patients entering the Cath-Lab should wear surgical face masks.
3. Doors must be closed, and no doors open between the Cath-Lab and the monitoring area.
4. The Cath-Lab staff should be divided into teams (A and B in our case) to limit the number needed to be isolated when confirming the contact with COVID-19 patients.
5. The radial approach is the access of choice because of less time of staff contact for homeostasis.
6. Transesophageal Echo (TEE) procedures were postponed as possible due to the high risk of infected droplet exposure.
7. All Acute Coronary Syndrome (ACS) patients planned for PCI were tested for COVID-19.
8. STEMI patients should undergo primary PCI whenever possible if it can be provided within an adequate time frame from the symptoms' onset and STEMI diagnosis.
9. After procedures on suspected or known COVID-19 patients, the need for final cleaning and sterilization has a top priority. Therefore, these cases were performed at the end of the working day, if possible.

## 5 Limitations and challenges

The recommendations detailed in this report are limited to our Cath-Lab facility during the COVID-19 pandemic; however, no clear guidelines are available to date on how to deal with this crisis. As well, the current COVID-19 PCR diagnostic test may not be sensitive enough to give clear feedback on the validity of our protocols. As well, the test was not routinely available during the lockdown at the beginning of the pandemic. Moreover, although we have advanced facilities, our resources are limited and, therefore, we applied and reported the best possible and suitable practice in response to this pandemic.

## 6 Summary

COVID 19 is a pandemic disease with high contagious rates that directly affected more than 46 million patients worldwide. The high transferable rates of this severe infection and the excessive mortality rates have overwhelmed the health care sector and have emplaced a significant burden on medical resources. This paper provides practical evidence in addition to expert opinion on the safe practice of cardiovascular medicine in Jordan. Our recommendations emphasize testing every patient with the acute coronary syndrome (ACS) for COVID-19 if possible or categorizing patients into low, intermediate, or high risk if testing is not available.
